# Ochratoxin A Management in Vineyards by *Lobesia botrana* Biocontrol

**DOI:** 10.3390/toxins5010049

**Published:** 2013-01-02

**Authors:** Giuseppe Cozzi, Stefania Somma, Miriam Haidukowski, Antonio F. Logrieco

**Affiliations:** Institute of Sciences of Food Production, National Research Council, Bari 70126, Italy; E-Mails: giuseppe.cozzi@ispa.cnr.it (G.C.); stefania.somma@ispa.cnr.it (S.S.); miriam.haidukowski@ispa.cnr.it (M.H.)

**Keywords:** *Lobesia botrana*, biological control, *Beauveria bassiana*, black aspergilli, ochratoxin A, grapes

## Abstract

Grape berries attacked by *Lobesia botrana* larvae are more easily infected by *Aspergillus *section *Nigri* (black aspergilli) ochratoxigenic species. Two-year field trials were carried out in Apulia (Italy) to evaluate a bioinsecticide control strategy against *L. botrana *and the indirect effect on reducing ochratoxin A (OTA) contamination in vineyards. A commercial *Bacillus thuringiensis* formulate and an experimental *Beauveria bassiana* (ITEM-1559) formulate were tested in two vineyards cultivated with the same grape variety, Negroamaro, but with two different training systems (espalier and little-arbor techniques). In both years and training systems the treatments by *B. bassiana* ITEM-1559 significantly controlled *L. botrana* larvae attacks with effectiveness similar to *B. thuringensis* (more than 20%). A significant reduction of OTA concentrations (up to 80% compared to untreated controls) was observed only in the first year in both training systems, when the metereological parameters prior to harvest were more favorable to the insect attack. Results of field trials showed that *B. bassiana* ITEM-1559 is a valid bioinsecticide against *L. botrana* and that grape moth biocontrol is a strategy to reduce OTA contamination in vineyard in seasons with heavy natural infestation.

## 1. Introduction

Ochratoxin A (OTA) is among the most harmful mycotoxins to pose a serious risk for human health as contaminants of several food commodities including wheat, oats, rice, grapes, raisins, wine, corn, soy, coffee, and beer. OTA is a very strong nephrotoxin and a renal carcinogen, with teratogenic and immunosuppressive properties and hepatotoxic, neurotoxic effects as assessed in several species of animals [1]. OTA was also associated with human Balkan Endemic Nephropathy and to Tunisian Nephropathy [2]. OTA is classified as a possible human carcinogen (group 2B) by the International Agency for Research on Cancer [3] and maximum levels in foods are regulated by the European Commission (EC No 123/2005).

Ochratoxin A contamination in grapes is mostly ascribed to *Aspergillus* section *Nigri* species (black aspergilli) [4]. The remarkable importance of these fungi, identified as the cause of OTA contamination in grapes, has been assessed in various geographical areas by several authors, such as Abarca *et al*. [5] in Spain, Sage *et al.* [6] in France, Da Rocha Rosa *et al.* [7] in Australia and Brazil, and by Leong *et al.* [8] in Argentina. Among the species belonging to *Aspergillus* section *Nigri*, *A. carbonarius* is considered the most important species due to its high percentage of ochratoxin-producing strains and due to its ability to produce high levels of OTA in a short time [1,4,9]. Black aspergilli are the cause of *Aspergillus* black rot of grapes, one of the many bunch rots occurring on grapes. Owing to their opportunistic behavior, the main route of infection is via damage to berry skin, caused by abiotic (rain, hail, wind, berry splitting) and/or biotic agents (grape berry moth, bunch mites) [4]. The moth *Lobesia botrana* (Denis & Schiffermueller) (Lepidoptera: Tortricidae) is a significant pest in the vineyards of southern Europe, where three or four generations per year occur, depending on environmental conditions in late summer [10]. The first-generation larvae cause damage to the inflorescences, while the second and third generations cause damage to unripe and ripening berries, respectively. A correlation between *L. botrana* occurrence and OTA contamination in grapes has been established [11,12,13]. In this respect, the control of *L. botrana* by using biocontrol agents is needed to increase healthy grape production with a low impact on environment. Biological control is also encouraged since the activity of entomopathogenic fungi and their natural metabolites is generally selective and no emerging resistance in pest populations is known, compared to synthetic insecticides [14].

The objective of this study was to determine whether biological control strategy against *L. botrana *larvae by two bioinsecticides could reduce OTA contamination in grapes. For this study, we used two biocontrol agents: a commercial formulate based on *Bacillus thuringiensis* and a strain of the entomopathogenic fungus *Beauveria bassiana* ITEM-1559 as a new candidate. 

## 2. Results and Discussion

**Figure 1:**
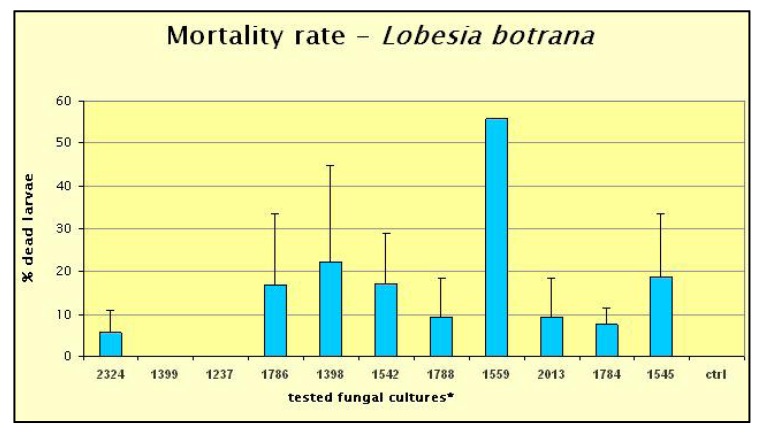
*Lobesia botrana *larva mortality in bioassays with different fungal formulates. Each bar shows the mean value with the standard deviation for 3 replicates. * tested fungal cultures: ITEM 2324—*Beauveria brongnartii*; ITEM 1786—*Paecilomyces fumosoroseum*; ITEM 1788—*B. bassiana*; ITEM 1784—*B. brongnartii*; ITEM 1399—*Fusarium subglutinans*; ITEM 1398—*F. semitectum*; ITEM 1599—*B. bassiana*; ITEM 1545—*B. brongnartii; *ITEM 1237—*Verticillium spp*; ITEM 1542—*B. bassiana*; ITEM 2013—*F. verticillioides*; Ctrl—control.

Among the strains isolated from insects in determining the mortality of *Lobesia botrana* larvae in the *in vitro* bioassay, no activity was detected for two strains, *Fusarium subglutinans* ITEM-1399 and *Verticillium* spp. ITEM-1237, while most of the other strains caused less than 20% larval mortality. *Beauveria bassiana* ITEM-1559, induced the highest mortality, with 55% of dead larvae, thus resulting the only effective fungal strain (Figure 1). 

In order to extend testing of its efficacy to environmental field conditions, two-year field trials were planned with *Beauveria bassiana* ITEM-1559, comparing it with a commercial pesticide based on *Bacillus thuringiensis*.

**Figure 2:**
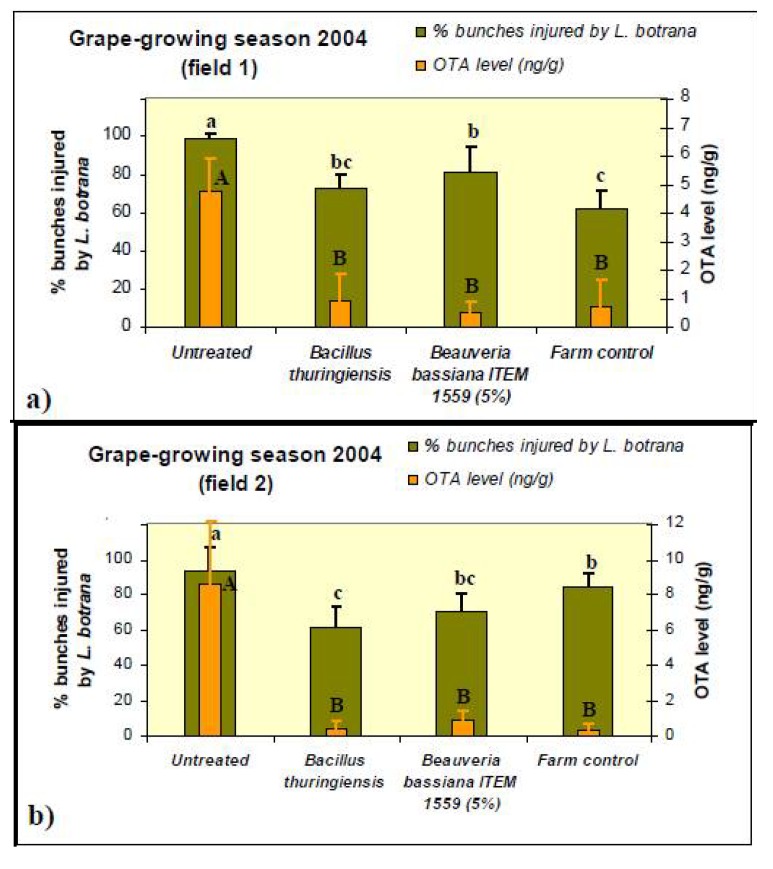
Incidence of diseased bunches and Ochratoxin A (OTA) content in four different treatments on the Negroamaro grape variety harvested with (**a**) espalier and (**b**) little-arbor training systems in 2004. Each bar represents the mean percentage of diseased bunches and the OTA level for each treatment. ANOVA on incidence of diseased bunches: field 1: *F*_(3,12)_ = 22.52, *p* < 0.001; field 2: *F*_(3,12)_ = 8.44, *p *< 0.01. ANOVA on OTA level: field 1: *F*_(3,12)_ = 20.35, *p* < 0.001; field 2: *F*_(3,12)_ = 19.17, *p* < 0.01. Mean values with the same letter were not significantly different (Duncan test, *p* ≤ 0.05).

In the field trials carried out during the 2004 grape-growing season (Figure 2), significant reductions (Duncan, *p* ≤ 0.05) in OTA levels, by up to 80% compared to the must samples of the untreated controls, were observed for all the treatments in both fields. Likewise, in both fields, the incidence of bunches injured by *L. botrana* larvae was significantly reduced by all treatments, compared to the untreated control. In particular, in field 1, treatments with *B. thuringiensis* (treatment 2) and chemical compound (treatment 4) showed the lowest values of moth larvae symptoms on bunches, without statistically significant differences from each other, as assessed by Duncan test (Figure 2a).

In field 2, treatments with *B. thuringiensis* (treatment 2) and *B. bassiana* ITEM-1559 (treatment 3) reported the lowest values of injured bunches, without any statistically significant difference from each other (Figure 2b). Moreover, the effectiveness of OTA reduction treatments was shown in both fields, as confirmed by the significant OTA reduction reported for all theses in which the incidence of bunches injured by *L. botrana* larvae was significantly reduced, (treatments 2, 3 and 4), compared to the untreated sample. However, no significant differences in OTA reduction were detected by Duncan test among treatments.

**Figure 3:**
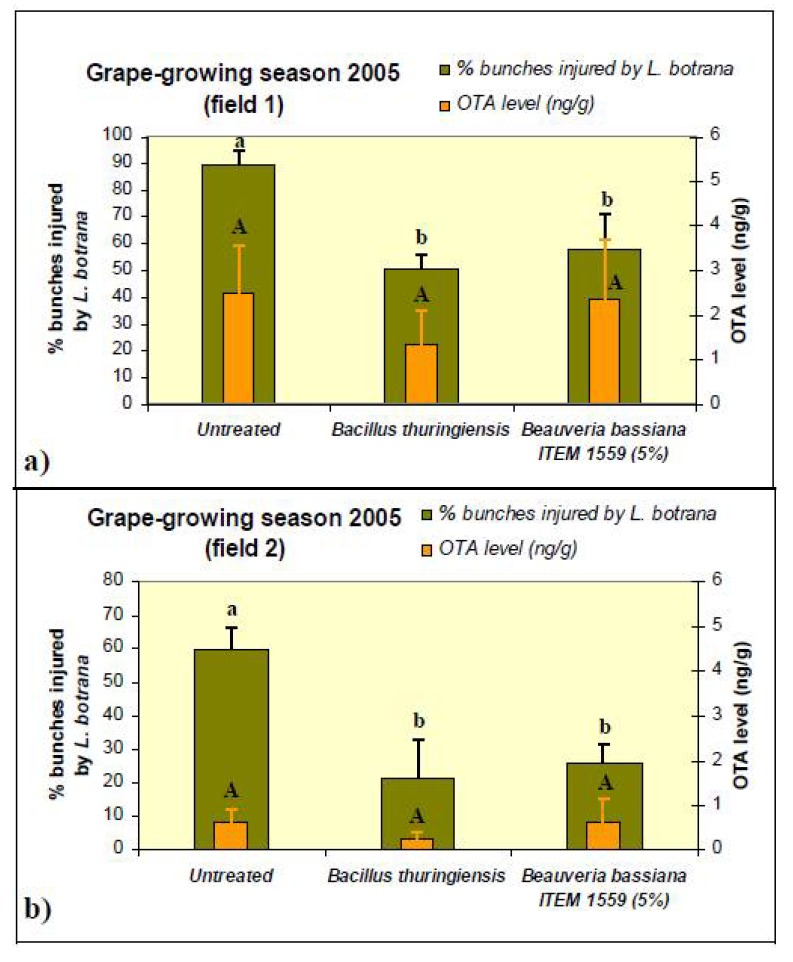
Incidence of diseased bunches and OTA content in four different treatments on the Negroamaro grape variety harvested with (**a**) espalier and (**b**) little-arbor training systems in 2005. Each bar represents the mean percentage of diseased bunches and the OTA level for each treatment. ANOVA on incidence of diseased bunches: field 1: *F*_(2,9)_ = 28.40, *p *< 0.001; field 2: *F*_(2,9)_ = 21.07, *p *< 0.01. ANOVA on OTA level: field 1: *F*_(2,9)_ = 1.39, *p *> 0.05; field 2: *F*_(2,9)_ = 1.19, *p *> 0.05. Mean values with the same letter were not significantly different (Duncan test, *p* ≤ 0.05).

In both fields in the 2005 grape-growing season (Figure 3), *B. thuringiensis* and *B. bassiana* ITEM-1559 significantly controlled the incidence of injured bunches (Duncan, *p* < 0.05), while no differences in OTA contamination among all the theses were observed (*p *> 0.05).

**Figure 4:**
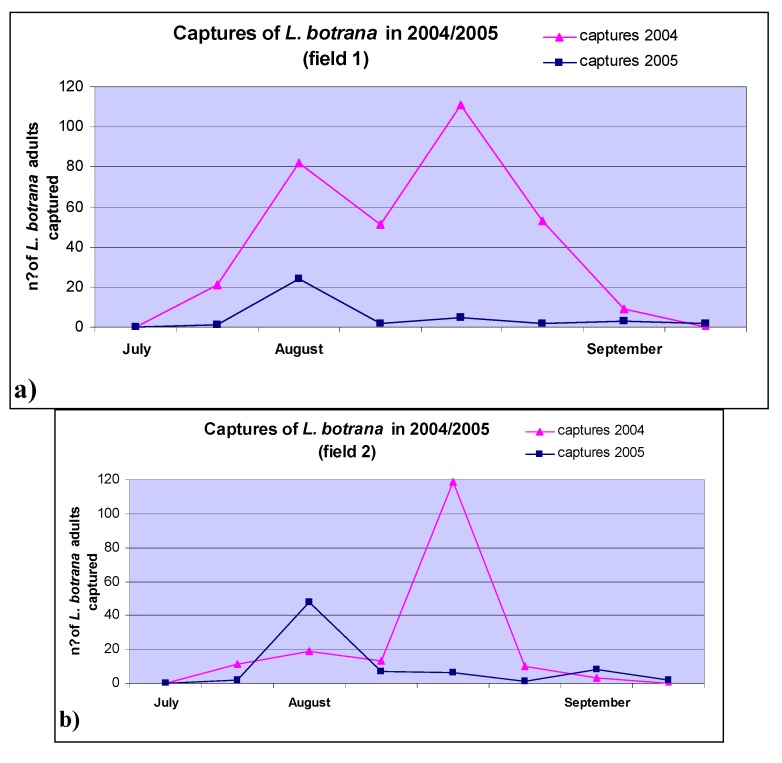
Captures of *Lobesia botrana* adults by pheromone traps in two-year experimental fields for biocontrol trials on the Negroamaro grape variety harvested with (**a**) espalier and (**b**) little-arbor training systems in 2004 and 2005.

Noteworthy, in the 2004 grape-growing season, more serious OTA contamination and *Lobesia botrana* occurrence were detected than in 2005. Moreover, as shown in Figure 4, the most relevant adult captures in 2004 occurred at late ripening (between August and September) [11,12], the phenological stage with the highest risk of black aspergilli contamination on grapes [15,16], especially when rain occurs prior to harvest and when the humidity in the vineyard is high. In this respect, higher rainfall and nocturnal relative humidity were registered in 2004 than 2005 by “Assocodipuglia” (Associazione Regionale Consorzi Difesa Puglia, [17]) meteorological stations.

The extremely low values of OTA contamination detected in the 2005 grape-growing season could have made less visible the differences among replicates, as well as among theses.

OTA production by black aspergilli is favored by warm weather conditions and rainfall, causing very high levels of contamination in the Mediterranean basin [4]. Other factors that have been found to affect OTA risk include agricultural practices, cultivar susceptibility and cropping systems [18]. In the present research, no influence of the training system used in the vineyard on *Lobesia botrana* occurrence and OTA contamination was observed, while other studies identified espalier as the cropping system with the highest OTA incidence [18].

However, our results confirmed that *L. botrana* larvae occurrence is a proven risk factor for OTA contamination in grapes, especially when the climatic conditions favor the infestation of the moth from the early veraison to the ripening stage. Control of the moth’s third generation is a crucial factor in reducing the injuries to bunches caused by the fourth-generation larvae, an important source of proliferation of black aspergilli.

Biological control strategies based on yeast strains have been proposed on grapes in order to reduce black aspergilli colonization and OTA accumulation [19,20]. Aiming to identify new useful biocontrol agents on grapes, the fungal strain *B. bassiana* ITEM-1559 showed significant control of *L. botrana* larvae in field conditions in two-year field trials, confirming the role previously established *in vitro*. In order to assess the safety of this strain in the field, the ability of *Beauveria bassiana* ITEM-1559 to produce beauvericin, a mycotoxin isolated for the first time from a *B. bassiana* strain (Bals) Vuill [21], was tested and proved to be negative (unpublished data). The entomotoxic activity of formulations based on *B. bassiana* ITEM-1559 gave similar results to *Bacillus thuringiensis*, which is known to be one of the most effective biocontrol agents for several pathogens [22]. However, further investigations could help to improve the efficacy of formulates containing the *B. bassiana* ITEM-1559 strain, that can now be regarded as a new candidate for biological control of *L. botrana*.

## 3. Experimental Section

### 3.1. Entomopathogenic Fungal Strains Selection

Eleven fungal strains isolated from insects, belonging to *Fusarium* (3 strains), *Beauveria *(6 strains), *Paecilomyces* (1 strain), and *Verticillium* (1 strains) genera, were obtained by ITEM microbial collection at ISPA-CNR [23] and used in *in vivo* bioassays on newborn larvae of *L. botrana* from captive-reared populations. 

Fungal cultures were obtained by growing the mycelium of each strain on 50 g of rice in 500 mL flasks, after incubation at 25 °C for three weeks. The cultures were then dried, ground and finally diluted in water to obtain concentrations ranging from 50 to 200 g/L. Formulate activity was tested by spreading 50 μL of each fungal suspension with a minimum concentration of 50 g/L on 16 larvae of *L. botrana*, in 128-well plates. Three replicates were performed for each fungal strain. After incubation at 26 °C for seven days, larva mortality rate was evaluated. 

### 3.2. Formulation with *B. bassiana* ITEM-1559 Preparation for Field Trials

*Beauveria bassiana* ITEM-1559 was selected on the basis of the larva bioassay as it showed the highest mortality (see Figure 1). Alive biomass of *B. bassiana* was produced on the modified Richard growing medium (KNO_3_ 10%; KH_2_PO_4_ 5%; MgSO_4_ 1.3%; sucrose 20%; FeCl_3_ 0.02%) in which 1 mL of microelements solution (3500 mg/L ZnSO_4_ּ7 H_2_O; 400 mg/L CuSO_4_ּ5 H_2_O; 280 mg/L MnSO_4_; 130 mg/L (NH_4_)_6_Mo_7_O_24_ּ4 H_2_O) was added. After incubation for 96 h in the BIOSTAT C fermenter (Braun Biotech Inc., Melsungen, Germany) under the following conditions: 25 °C (±0.5 °C), 25% of O_2_ percentage, pH 6, the count of *B. bassiana* ITEM-1559 colonies was 2 × 10^8^ CFU/mL. The *B. bassiana* culture was then stored for a week at 4 °C until use.

### 3.3. Field Trials

Two-year trials (2004 and 2005) for the biological control of *Lobesia botrana* were carried out in two vineyards of Apulia, in southern Italy. As well as a commercial bioinsecticide based on *Bacillus thuringiensis*, the efficacy of a formulation of *Beauveria bassiana* ITEM-1559 was tested. In both years, the field trials were carried out in two vineyards cultivated with the same grape variety, Negroamaro, that is one of the most widely cultivated grape varieties in Apulia, characterized by a high cluster compactness that contributes to the susceptibility of this cultivar to *L. botrana* larvae infestation [24] and to *A. carbonarius* and OTA contamination [25]. Vines were cultivated with two different training systems in the two fields; in particular, the espalier technique in field 1 and the little-arbor technique in field 2. Plant density and raw spacing were, respectively, 0.38 plants m^−2^ and 1.3 m in the field 1, and 0.42 plants m^−2^ and 1.2 m in the field 2. For each experimental trial, a randomized block design on four rows was adopted, with four plots (replicates) per treatment, each including 15 grapevines. In 2004 field trials, four plots in each row were identified, for a total of 60 plants per row. On the other hand, in 2005, field trials were conducted on 45 plants per row, since only three treatments were planned. The following treatments were compared: an untreated control (treatment 1), a treatment with *B. thuringensis* (treatment 2), a treatment with *B. bassiana* (treatment 3) and a farm control with a conventional insecticide protocol (treatment 4). In particular, 70 g/hL of a commercial formulate containing 6.4% *Bacillus thuringiensis* Berliner, var Kurstaki (serum-type 3a, 3b, strain SA 11), with an insecticide activity of 53,000 SU/mg (SU = Spodoptera Units, based on *Spodoptera exigua*), was used in treatment 2. In treatment 3, a suspension at 5% of a *B. bassiana* culture with 2.0 × 10^8^ CFU/mL was utilized. For treatments 2 and 3, the application of formulates started with the first capture of adults on pheromone traps in the field (coinciding with the beginning of the early veraison on bunches) and were carried out once a week until a week before the grape harvest. By contrast, a control protocol with the insecticide chlorpyrifos was used as conventional control only in the 2004 trials for treatment 4. Chemical experimental protocol with chlorpyrifos and the time of insecticide application were based on crop stage and *L. botrana *infestation. In particular, 52.5 g/hL of a 75% clorpyrifos formulation were applied on the grapes at three different *L. botrana* and crop stages: (1) at the 1st instar larvae of I carpophagous generation (grape setting); (2) when eggs of carpophagous generation appear (before bunch closing); (3) at the 1st instar larvae of II carpophagous generation (veraison).

### 3.4. Lobesia Botrana

*Lobesia* *botrana* adult occurrence was monitored using sex pheromone traps. The effects of the *L. botrana* control strategy were evaluated at harvest. Each bunch was visually inspected and the occurrence of bunches with almost one berry injured by the grape moth larvae was observed in about 100 bunches per plot (400 bunches per treatment). Then the incidence of bunches damaged by *L. botrana* (expressed in percentage), was calculated for each treatment.

### 3.5. Sampling of Grapes

At the grape harvest, the whole grape production was collected, except for the border plants in each plot. The grape samples were crushed during the day by using a motorized crusher de-stemmer (“mod. Ares 15” single phase motor 230V/50HZ—HP 1). The crushed grape samples (1 kg for each replicate) were refrigerated at 20 °C and processed to determine the OTA level. 

### 3.6. Determination of Ochratoxin A (OTA)

OTA was measured as described by Visconti *et al.* [26] with minor modifications. The must sample was centrifuged at 4000 rpm for 10 min at 4 °C and 10 mL volume of centrifuged sample was then diluted with a 10 mL water solution containing PEG (1%) and NaHCO_3_ (5%), mixed and filtered through a Whatman GF/A glass microfibre filter. A 10 mL volume of diluted extract was cleaned up through an OchraTest (Vicam, Milford, MA, USA) immunoaffinity column at a flow-rate of about 1 drop per second. The column was washed with a 5 mL solution containing NaCl (2.5%) and NaHCO_3_ (0.5%) followed by 5 mL distilled water at a flow-rate of 1–2 drops per second. OTA was eluted with 1.5 mL methanol and collected in a silanized clean vial (Kimble Chase, Vineland, NJ , USA). The eluted extract was evaporated under a nitrogen stream at *ca.* 50 °C and reconstituted with 500 μL of the HPLC mobile phase. A 100 μL of reconstituted extract was injected into the HPLC apparatus Agilent Technology Series 1100 (Agilent Technologies Inc., Santa Clara, CA, USA) with a full loop injection system. The fluorometric detector was set at wavelengths, ex = 340 nm, em = 460 nm. The analytical column was Zorbax SB-C_18_ 4.6 × 150 mm, 5 μm with a guard column inlet filter (0.5 μm × 3 mm diameter; Rheodyne Inc., Rohnert Park, CA, USA). The mobile phase consisted of a mixture of acetonitrile-water-acetic acid (99:99:2, *v*/*v*/*v*) at a flow rate of 1 mL/min. OTA was measured by comparing peak areas with a calibration curve obtained with OTA standard solutions. The detection limit was 0.01 ng/mL.

### 3.7. Statistics

The results of the incidence of diseased bunches and of OTA content showed in Figure 2 and Figure 3, are the mean of four replicates for each treatment. Percentage data of bunches injured by *L. botrana* were arcsine transformed before statistical analyses, though the original nontransformed percentage data are showed in the figures. The results obtained were previously analysed by ANOVA test, then the Duncan test was used to compare the means (*p *≤ 0.05). All statistical analyses were performed with the statistical software package for Windows STATISTICA version 6.0 (StatSoft, Tulsa, OK, USA).

## 4. Conclusions

*Lobesia botrana* is confirmed to be an important ochratoxin A risk factor for grapes in the field, during seasons with heavy natural infestation. Effective biocontrol of the last generation of moth larvae is useful to reduce indirectly the health hazards of OTA. The entomopathogenic fungal strain of *Beauveria bassiana*, ITEM-1559, showed high entomotoxic activity on *L. botrana* larvae of the third generation, and can thus be regarded as a good candidate in biocontrol in the vineyard. 
